# Fast-Track to Protection? A Review of Encepur’s Express Dosing Schedule for Tick-Borne Encephalitis

**DOI:** 10.3390/v17111439

**Published:** 2025-10-29

**Authors:** Kyra Zens, Ralph Torgler, Michael Horn, Carsten Schade Larsen

**Affiliations:** 1 Epidemiology, Biostatistics and Prevention Institute, University of Zurich, CH-8001 Zurich, Switzerland; zens@immunology.uzh.ch; 2 Institute for Experimental Immunology, University of Zurich, CH-8057 Zurich, Switzerland; 3 Bavarian Nordic Berna GmbH, Oberriedstrasse 68, CH-3174 Thoerishaus, Switzerland; 4 Kinder- und Jugendarztpraxis, Achenweg 1, 83471 Schönau, Germany; 5 Department of Infectious Diseases, Aarhus University Hospital, Palle Juul-Jensens Boulevard 99, DK-8200 Aarhus N, Denmark

**Keywords:** tick-borne encephalitis, TBE vaccine, accelerated vaccination schedule, safety, immunogenicity

## Abstract

Cases of confirmed tick-borne encephalitis (TBE) have increased dramatically over the last 30 years, highlighting growing endemicity across Eurasia. Two preventative vaccines, Encepur^®^ (Bavarian Nordic A/S, Hellerup, Denmark) and FSME-Immun^®^ (Pfizer Ireland Pharmaceuticals, Cork, Ireland), are licensed in Europe. For both vaccines, primary immunisation consists of a three-dose regimen, administered over approximately one year using “Conventional” dosing schedules. Both vaccines can also be administered using “Rapid” schedules, which shorten the interval between the first two doses but still take around a year to complete. Currently, only Encepur offers an approved “Express” schedule, whereby all three priming doses are given within 21 days. The effectiveness of TBE vaccination is markedly higher in individuals who receive ≥3 doses, compared with those who receive only one or two doses, indicating the importance of series completion. Moreover, seropositivity takes several weeks to develop after vaccination. As such, individuals are advised to initiate vaccination before peak tick season to allow sufficient time to develop protective immunity during periods of highest risk. Despite these considerations, vaccine uptake and series completion remain suboptimal in TBE-endemic regions. Furthermore, many vaccinees—including travellers with limited time before departure and residents of endemic areas—do not initiate vaccination until peak tick season, when risk is greatest. Broader use of Encepur’s Express schedule may help to address these challenges. The Express schedule’s 21-day timeframe may help to increase series completion by reducing drop-offs associated with prolonged dosing intervals. Additionally, it can support timely protection by enabling series completion, with sufficient time post-vaccination to develop protective immunity, all within a single-risk season, even among late initiators. In this narrative review, we evaluate the safety and immunogenicity of Encepur’s Express schedule and discuss its potential utility across a broader range of vaccinees. These insights may help inform TBE vaccine recommendations and support efforts towards improving vaccination strategies amid increasing TBE risk.

## 1. Introduction

Tick-borne encephalitis (TBE) is a potentially life-threatening, vector-borne neurological disease caused by infection with the TBE virus (TBEV) [1]. TBEV infections are transmitted to humans predominantly via the bite of infected ticks [2], typically during outdoor leisure activities, such as walking, hiking or camping [3,4]. Individuals who work outdoors, such as forestry workers or farmers, or those living in rural environments, are also at elevated risk [3,4,5]. Due, in part, to improvements in diagnostic capabilities, surveillance, and reporting [6,7], confirmed cases of TBE have increased by approximately 400% over the past 30 years [5], making it the second-most common tick-borne disease in Europe after Lyme borreliosis. TBEV is now endemic in 27 European countries, with several additional countries across Eurasia considered “at risk” [8,9]. In Europe alone, an estimated 12,000 cases of TBE occur annually [10,11]; however, true figures are likely substantially higher due to underreporting of mild and asymptomatic cases [8,12,13].

Despite the growing extent of TBEV endemicity and the potential severity of TBE, no specific curative therapies are currently available, making effective disease prevention critical [14]. In Europe, two vaccines are approved for use in adults and children: Encepur^®^ (Bavarian Nordic A/S, Hellerup, Denmark) and FSME-Immun^®^ (Pfizer Pharmaceuticals, Cork, Ireland [also licensed as TicoVac^®^]) [15]. Both are inactivated formulations based on highly homologous strains of the European TBEV subtype; namely K23 (Encepur), and Neudoerfl (FSME-Immun) [16,17,18,19]. Both vaccines may be administered according to “Conventional” dosing schedules consisting of three priming doses administered over approximately one year, as well as “Rapid” schedules with shortened intervals between the first two doses but with similar total duration (Table 1). Encepur may additionally be administered according to an “Express” schedule, in which all three priming doses are administered over 21 days. For both vaccines and all available schedules, regular boosters are required to maintain lasting protection. Both vaccines are generally considered to be interchangeable, meaning that the primary immunisation series can be initiated with one vaccine and completed with the other without compromising immunogenicity [20]. However, current data on interchangeability are principally derived from studies using Conventional dosing regimens or from those where the dosing schedule is not explicitly stated, meaning that vaccine interchangeability when using the Express schedule remains to be confirmed.

Irrespective of dosing schedule, completing the full three-dose primary series is critical to achieving optimal protection [21,22,23,24,25,26,27,28]. Indeed, real-world evidence suggests that TBE vaccines are 89–99% effective in individuals who receive ≥three doses [21,22,23,24,25,26,27,28], whereas vaccine effectiveness (VE) is substantially lower in individuals receiving only one or two doses [22,23,28]. Despite this, TBE vaccine uptake remains low across many TBE-endemic regions in Europe and vaccine compliance is suboptimal, with a substantial fraction of vaccinees failing to receive all three recommended priming doses [27,29,30,31,32].

From an immunological perspective, the timing of vaccination is also an important consideration. TBE vaccine recommendations generally advise initiating the primary series in winter to allow for the administration of at least two doses prior to Spring and Summer, when tick activity and subsequent risk of TBEV infection peaks [33,34]. Ideally, the primary series should be completed at least one month prior to exposure, where possible [35], to allow sufficient time for seroconversion prior to exposure risk. However, many vaccinees, whether travellers or residents of TBE-endemic countries, do not initiate vaccination until shortly before potential exposure, resulting in incomplete immunisation and suboptimal protection.

Collectively, these observations highlight the need to improve TBE vaccine compliance and to identify vaccination strategies that can provide protective immunity as quickly as possible. In this review, we specifically discuss the safety, immunogenicity, boostability and durability of Encepur’s unique Express schedule. We further outline scenarios where the use of this schedule may be preferable to ensure timely protection of at-risk individuals. Together, these insights are intended to support informed decision-making among healthcare professionals (HCPs) and policymakers to optimise TBE prevention strategies.

## 2. TBE Vaccine Dosing Schedules

Both Encepur and FSME Immun are available in adult and paediatric formulations (Encepur Children and FSME Immun Junior) and can be administered to all individuals from one year of age at risk of TBE [16,17,18,19]. Primary dosing schedules for corresponding adult and paediatric formulations are the same for both vaccines, as summarised in Table 1.

**Table 1:** Current TBE vaccine dosing schedules for primary immunisation.

	Encepur			FSME-Immun		
Primary Dosing Schedule	1st Dose	2nd Dose	3rd Dose	1st Dose	2nd Dose	3rd Dose
Conventional	Day 1	1–3 months ^a^	9–12 months ^b^	Day 1	1–3 months ^a^	5–12 months ^b^
Rapid ^c^	Day 1	Day 14 ^a^	9–12 months ^b^	Day 1	Day 14 ^a^	5–12 months ^b^
Express ^d^	Day 1	Day 7 ^a^	Day 21 ^b^	-	-	

^a^ After first primary dose; ^b^ After second primary dose; ^c^ Formerly known as “Accelerated Conventional” for Encepur; ^d^ Formerly known as “Rapid” for Encepur. Both vaccines are recommended as a three-dose regimen for primary immunisation. “Conventional” and “Rapid” schedules are available for both Encepur and FSME-Immun; however, the “Express” schedule is unique to Encepur.

As described, both vaccines can be administered according to Conventional or Rapid schedules [16,17,36]. With Conventional schedules, doses are given at Day 0, 1–3 months, and either 5–12 months for FSME-Immun or 9–12 months for Encepur [16,17] (Table 1). Rapid schedules for either vaccine follow Conventional schedules for both the first and third doses, but the second dose is administered at Day 14 (Table 1). With Encepur’s Express schedule, however, all three priming doses are administered over the course of 21 days (Day 0, Day 7, and Day 21) [16,37] (Table 1).

As aforementioned, continued protection following primary TBE vaccination requires periodic booster vaccination. The recommended schedules for booster doses are summarised in Table 2. For both vaccines, individuals vaccinated according to Conventional and Rapid schedules should receive a first booster three years after the final dose of the primary series. With Encepur Express, a first booster should be administered 12–18 months after completing the primary schedule. Regarding subsequent boosters, for FSME-Immun these should be administered every five years in those aged 16–60 years and every three years in those over 60 years, when using either of the primary dosing schedules [36]. For Encepur, on the other hand, subsequent boosters should be administered every five years in those <12 years old, every 5–10 years for those aged 12–49, and every three years in those >49 years old, irrespective of primary schedule [16,17] (Table 2).

## 3. Safety Profile with Encepur Express

Encepur and FSME Immun have been licensed since 1991 and 1976, respectively [17,18]. Both vaccines have well-established and well-studied safety profiles and are generally considered to be well-tolerated [17,18]. In a systematic review of available safety data, Rampa et al. found that the safety profile was comparable between both vaccines, as well as across all available vaccination schedules [20]. Based on pooled data, 25% of vaccinees reported local reactions or mild adverse events, most commonly including injection-site pain and local swelling, irrespective of vaccine type or dosing schedule [20]. Systemic reactions, which most commonly included fever and malaise, were reported by 30% of vaccinees (1–46%); however, the frequency of these reactions decreased with successive vaccine doses [20]. Serious adverse events were extremely rare with either vaccine or any dosing schedule [20].

Focusing solely on Encepur’s Express schedule, cumulative findings from multiple randomised clinical trials involving approximately 4000 adults, adolescents and children demonstrate a safety profile comparable to the overall safety findings for Encepur [38,39,40,41]. Consistent with this, there have been no reports of any vaccine-related serious adverse events with the Express dosing schedule among adults or children, nor has the Express schedule been associated with any increased safety concerns in clinical trials thus far [38,39,40,41]. Several studies have also reported safety findings following the administration of a first booster 12–18 months after completion of the Express primary schedule, or subsequent boosters 3–5 years later [37,42,43]. In these studies, adverse events were infrequent and generally mild, reinforcing Encepur’s long-term safety profile [37].

## 4. Immunogenicity of Encepur Express

A definitive correlate of protection following TBE vaccination has not yet been established. While it is important to bear in mind that the full picture of protective immunity is likely complex [44], TBEV-specific neutralising antibody titres at a level ≥ 10, as measured by the neutralisation test (NT), have been widely used as a surrogate marker of protection against TBE infection, including by the World Health Organization (WHO) [45,46]. This threshold was also used in relevant licensure studies of both Encepur and FSME-Immun [47].

**Figure 1:**
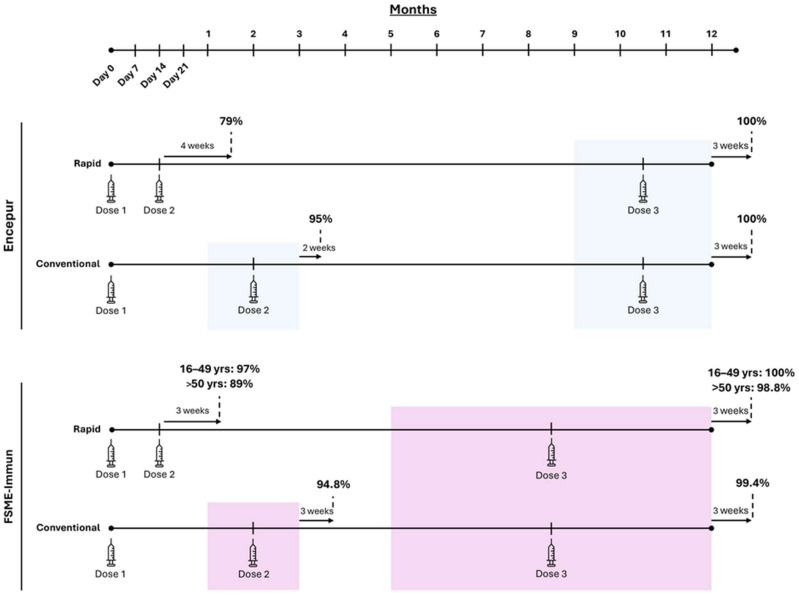
Timescale of seroconversion with either the Rapid or Conventional dosing schedules for Encepur or FSME-Immun. Data shown as the proportion of vaccinees with TBEV-specific neutralising antibody titres ≥ 10 after each dose [16,17,18].

The immunogenicity of Encepur and FSME-Immun has been extensively evaluated, with approximately 40 clinical studies assessing immune responses across all dosing schedules. Previous studies indicate that completion of the primary series for either vaccine results in seroconversion (NT ≥ 10) in approximately 100% of vaccinees, irrespective of Rapid or Conventional dosing schedules [17,18] (Figure 1). Using Conventional dosing for either vaccine, approximately 95% of adults seroconverted within three weeks of receiving a second dose [17,18] (Figure 1). With Rapid dosing, the immune response following a second dose was slightly more variable, with 79% of vaccinees seropositive four weeks after the second dose of Encepur [17], and 89–97% of vaccinees seropositive three weeks after their second dose of FSME-Immun, depending on age [18] (Figure 1). Paediatric data show similarly robust immune responses, with 98–100% of children demonstrating seropositivity 2–3 weeks after a second or third dose of either vaccine using the Conventional dosing schedule [16,19]. Comparable findings have been shown with Encepur’s Rapid schedule, though corresponding data are not currently available in children for FSME-Immun’s Rapid schedule [16,19].

**Figure 2:**
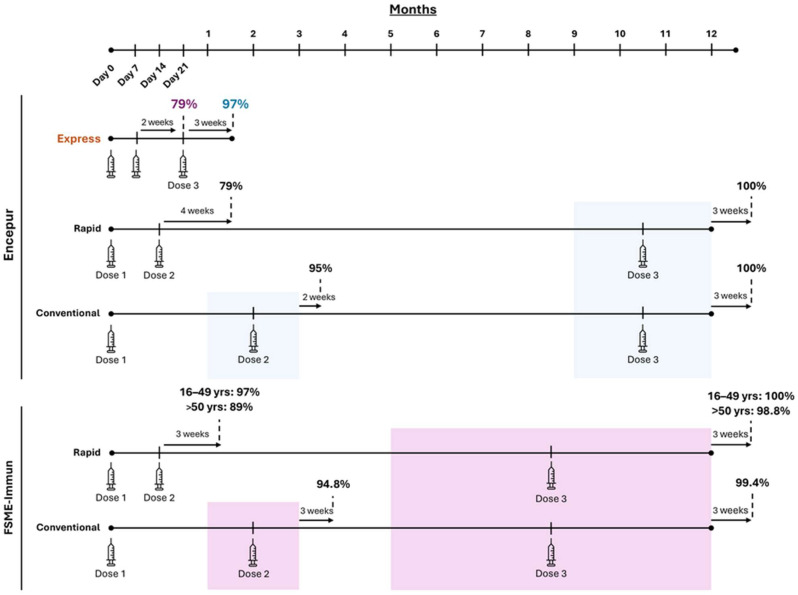
Timescale of seroconversion with all dosing schedules for Encepur or FSME-Immun. Data shown as the proportion of vaccinees with TBEV-specific neutralising antibody titres ≥ 10 after each dose [16,17,18].

With Encepur’s Express schedule, 97% of vaccinees were seropositive three weeks after completing the primary series, consistent with seroconversion rates following completion of either vaccine’s Rapid or Conventional schedules [17] (Figure 2). Similarly, approximately 100% of children seroconverted after completing the Express schedule [16].

While the above findings demonstrate robust immune responses after two doses with both Conventional and Rapid TBE vaccination schedules, these responses still require 2–3 months from the initiation of the vaccine regimen to develop. Moreover, the maximum response, in terms of the proportion of seropositive vaccinees, is only achieved after the third dose of either vaccine, which may be administered no earlier than 5–12 months after the first dose (9–12 months for Encepur) (Figure 1). With Encepur Express, however, the maximum threshold of protection can be achieved within just 1–2 months of initiating vaccination, since all three doses can be administered within a 21-day window (Figure 2). Previous reports have demonstrated that antibody persistence prior to a first booster is maintained for at least 300 days after primary vaccination with the Express schedule [40]. These findings indicate that the Express schedule provides both a rapid and sustained immune response, supporting its suitability for both short-term travellers requiring fast protection and residents seeking durable immunity.

### Clinical Evidence to Support the Immunogenicity of Encepur Express

A more detailed analysis of key studies describing the immunogenicity of Encepur’s Express schedule further demonstrates its capacity to induce a rapid and robust immune response across diverse age groups. In a 2003 study, Zent et al. reported immunogenicity data from a total of 3118 subjects, aged 12–76 years old, taken from three Phase III clinical trials [39]. All vaccinees seroconverted (defined as the development of TBEV-specific neutralising antibodies in seronegative subjects, or a ≥4-fold increase in neutralising antibody titres for seropositive subjects) approximately three weeks after completing the primary dosing regimen [39]. Additionally, all 114 adolescent participants had TBEV-specific neutralising antibodies at Day 21, indicating an onset of seroconversion prior to administration of the third dose [39]. Although designed as a non-inferiority trial for a new vaccine formulation, this study provides evidence of rapid and high immunogenicity associated with the Express schedule.

Thereafter, Schöndorf et al. assessed the immunogenicity of Encepur in 398 healthy subjects, aged 12–64 years, using four different primary dosing schedules: the Express Schedule, the Rapid schedule, the Conventional schedule, and a Modified Conventional schedule, whereby the second dose in the primary series was administered at Day 21 [40]. By Day 21, geometric mean titres (GMTs), as measured by NT (NT-GMTs), were highest among individuals receiving the Express or Rapid dosing schedules, likely due to the administration of two or more doses by this timepoint [40]. Similarly, at Days 42, 180, and 300, NT-GMTs were highest among those receiving either the Express or Conventional dosing schedules, reflecting completion of the Express schedule at the earliest timepoint and the administration of the second and third doses of the Conventional regimen at the later timepoints, respectively [40]. These data highlight the timeliness of protection conferred by Encepur Express in comparison to the other dosing schedules, which is an important consideration for those requiring accelerated immunity.

The Express dosing schedule has also been widely studied in paediatric populations. In a 1996 study to determine the paediatric dose for Encepur, immunogenicity was assessed among 522 healthy children, aged 18 months to 14 years. Participants were assigned 1:1:1 to receive either 0.4 µg, 0.75 µg, or 1.5 µg of Encepur according to the Express dosing schedule [38]. The comparator group consisted of adults aged 18–60 years, receiving a 1.5 µg dose via the Express schedule [38]. Post-vaccination GMTs, as measured by enzyme-linked immunosorbent assay (ELISA), increased with increasing dose; however, there was an overall decrease observed with increasing age among children [38]. In children < 12 years of age who received 0.4 or 0.75 µg doses, GMTs measured by ELISA were equivalent to those observed in adults with the standard dose (1.5 µg) [38]. Conversely, GMTs among children < 12 years old who received the standard dose were higher than those in adults, whereas titres in 12–14-year-olds were comparable to adult levels [38]. Nonetheless, 99–100% of children, across all age groups, and 99% of adults seroconverted, as defined by ≥50% virus neutralisation in the NT using a high-challenge dose of virus (100× TCID_50_) [38].

Similarly, in a 2007 Phase III randomised trial, Schöndorf et al. compared immune responses in children aged 1–11 years following vaccination with ‘Encepur Paediatric’ (0.75 µg) according to the Express, Conventional, and Modified Conventional schedules used in their earlier study [41]. By Day 42, 97–100% of participants were seropositive (NT ≥ 10), regardless of dosing schedule. However, while the proportion of seropositive individuals was maintained up to Day 300 in the Express group, it declined to 90% and 86% with the Conventional and Modified Conventional schedules, respectively [41]. By Day 300, NT-GMTs were significantly higher among children who had completed the Express group, versus those who had only received the first two doses of the Conventional or Modified Conventional schedules, reflecting the faster onset of optimal protection due to expedited dosing and series completion [41].

Overall, these findings support the conclusion that Encepur’s Express primary immunisation schedule provides rapid seroprotection to vaccinees across all age groups, inducing stable antibody titres for at least 300 days after vaccination [38,39,40,41].

## 5. Booster Response with Encepur’s Express Schedule

While seropositivity following TBE vaccination can persist for 10 or more years after completion of the primary series, TBEV-specific NTs gradually decline over time, particularly in vaccinees ≥ 50 years old [48,49,50]. Without a correlate to protection, it is difficult to deduce the implications of this phenomenon on long-term immunity; however, these data generally support the administration of at least one booster dose. In a cross-sectional study, Lindblom et al. reported that the proportion of seropositive individuals increased from 59% among those with three doses, to 84–96% among those with four or five doses, respectively [49]. Notably, NTs in older vaccinees (≥60 years old) who had received a fourth dose were comparable to titres in younger vaccinees after three doses, highlighting the importance of additional vaccine doses, particularly in the older population [49].

The boostability of Encepur and FSME-Immun have been well studied across more than 10 clinical trials [20,42,51]. Overall, the evidence available suggests that booster responses are consistently strong and are comparable between vaccine brands and across primary dosing schedules [20,42,51,52]. Nonetheless, with growing interest in accelerated vaccination strategies, detailed evaluation of long-term outcomes following the Express schedule remains an important consideration.

Indeed, in a follow-up to their initial immunogenicity and safety study, Zent et al. assessed responses in 222 adults aged 19–51 years who received a first booster 12–18 months after primary vaccination via the Express schedule [37]. Prior to receiving a booster, all but one participant classed as seronegative (NT < 2) still had detectable NTs, despite an approximately 20% decline in NT-GMTs in the time since completing the primary series. Nonetheless, a robust anamnestic response was observed after boosting, with a 43-fold increase in GMTs by Day 21 [37]. In contrast, participants who were seropositive prior to receiving a first booster demonstrated a more modest rise in post-booster NT-GMTs, consistent with an early booster-like response following primary immunisation and sustained high antibody levels thereafter [37].

Subsequent follow-up studies where primary immunisation was completed via the Express schedule confirm the durability of this booster response. For example, Beran et al. reported that all individuals remained seropositive three years after receiving a first booster 12–18 months after primary immunisation. GMTs of TBE-specific antibodies, as measured by ELISA, remained well above the level of detection prior to administration of a second booster three years later [42]. Furthermore, the administration of the second booster resulted in a rapid and robust immune response [42]. The same group also provided evidence of sustained seropositivity over longer periods following the administration of a booster dose after completion of the primary series via the Express schedule [43,53,54]. In a 5-year follow-up study of 323 individuals aged≥15 years, Beran et al. reported that ≥94% of individuals were seropositive at all timepoints up to Year 5 after receiving a booster [43]. Similarly, in their study evaluating responses 6–10 years after receiving a first booster, >97% of individuals remained seropositive at Year 10, regardless of primary dosing schedule [54]. Beran et al. also conducted a 15-year follow-up study in individuals who received their first booster 12–18 months after completing the Express schedule, noting that 100% of participants remained seropositive at all timepoints up to Year 15, including those over 60 years of age [53].

Together, these findings demonstrate robust immune responses and long-term seropositivity for up to 15 years following the administration of Encepur’s Express primary dosing schedule coupled with appropriately timed boosters [53].

## 6. Real-World Estimates of VE for TBE Vaccines

At present, there are no published real-world data specifically reporting VE associated with Encepur’s Express schedule; however, available evidence strongly supports the effectiveness of both licensed TBE vaccines (FSME-Immun and Encepur) across dosing schedules. Multiple large population-based studies have reported VE estimates ranging from 89% to 99% after completion of at least the three-dose primary immunisation series, including among children, as well as older individuals [21,22,23,24,25,26,27,28].

However, while partial vaccination conveys some protection against TBE, estimates of VE are generally lower among individuals who received only one or two doses, compared with those who completed the primary series [22,23]. Indeed, Zens et al., reported VE of 77% for individuals who received only one or two TBE vaccine doses [23], while Nygren et al. reported VE of 78% specifically in those who had received one dose and 85% in those who had received two doses [22]. In the aforementioned studies, estimates of VE increased to 95% and 97%, respectively, among fully vaccinated individuals, highlighting the importance of completing the full primary series to achieve optimal protection. In this context, the expedited timing of the Express schedule may play a particularly important role in maximising VE by supporting completion of the full three-dose series within a shorter and more manageable timeframe, potentially reducing the likelihood of drop-offs between doses.

While Erber et al. did not observe a notable difference in VE following two versus three doses of TBE vaccine, estimating 92–93% VE for either number of doses among their German cohort and 97–99% in their Latvian cohort [21], they did find that VE increased to 95% in the German cohort and remained around 99% in the Latvian cohort following a booster (fourth dose) [22]. Similarly, Nygren et al. reported that VE increased from 92% after three doses to approximately 96% after receiving at least one booster, irrespective of age [22].

Additionally, several studies have demonstrated that VE in fully vaccinated individuals remains high for at least 10 years or more following the last vaccine dose [22,23,28]. In line with these findings, and based on a combination of long-term seropositivity data from clinical studies and VE data demonstrating prolonged protection in real-world settings, Encepur has had a recent label change to allow for subsequent 10-yearly boosters for individuals 12–49 years of age, following completion of the primary vaccination series and the administration of a first booster.

## 7. Future Considerations for Broader Use of the Express Schedule

In light of the increasing burden of TBE despite widespread vaccine availability, there is a need to increase awareness of alternative dosing regimens that have the potential to boost vaccine coverage, thereby improving disease prevention efforts. Thus far, the wider application of expedited primary immunisation schedules for TBE has been largely overlooked. In this regard, Encepur’s Express schedule is usefully positioned to allow vaccinees to achieve lasting protection on an expedited timeline, making it a practical option for broader integration into routine immunisation strategies across all age groups. In countries without a reimbursement policy for TBE vaccination, the timing of expenditure should also be considered an important factor when making recommendations. For example, the Encepur Express schedule requires the completion of three priming doses and a first booster dose within a 12–18-month window, whereas the Conventional dosing schedule sees delivery of the three-dose primary series and first booster over a 3–4-year timeframe. In both scenarios, total vaccine costs would be the same; however, the latter strategy would allow for costs to be spread out over a longer period of time, which may be an important consideration among some vaccinees.

### 7.1. Travel-Specific Needs

Ideally, international travellers are recommended to have a medical consultation at least six weeks prior to their departure, partly to ensure sufficient time to administer multi-dose vaccines, such as the TBE vaccine [55,56,57]. However, studies have demonstrated that travellers typically present to a travel clinic a median of 3–4 weeks prior to departure, with a quarter of travellers doing so within two weeks of departure (so-called “last minute travellers”) [58,59]. In such scenarios, individuals may not have sufficient time to complete even the first two doses of the primary immunisation series according to Conventional or Rapid schedules. Conversely, some travellers may initiate TBE vaccination in ample time for Conventional or Rapid dosing but with the intention of only receiving two doses, which likely provides only short-term protection. Using the Express schedule in either of these scenarios offers the advantage of enabling vaccinees to complete the full three-dose series within the same pre-travel timeframe, thereby providing both immediate and longer-term protection. This is particularly beneficial for those who travel frequently to TBE-endemic areas or may be at risk of future exposure to TBE. Routinely incorporating the Express schedule into travel medicine protocols may not only improve flexibility for short-notice travel but also promote greater awareness of this schedule among HCPs.

### 7.2. Residents of TBE-Endemic Regions

Among residents of TBE-endemic areas, low perception of risk, vaccine complacency, and suboptimal public awareness may contribute to poor vaccine uptake or delayed initiation of the vaccination series [32]. As described, the optimal timing for initiating TBE vaccination is in the winter months, when TBEV risk is low [33,34]. This allows for at least two doses of TBE vaccine to be administered before the high-risk period when using Conventional or Rapid schedules. However, individuals who postpone initiating vaccination until the spring or summer months, just as tick activity and subsequent TBE risk increase, leave themselves insufficient time to achieve adequate protection before peak exposure. The Express schedule offers a strategic and immunological advantage in this seasonal context. It enables late initiators to not only complete the primary series, but also to mount a sufficient immune response, which peaks around three weeks after receiving the final dose, all within a single-risk season. Integrating the Express schedule into national vaccination campaigns or seasonal outreach initiatives could promote timely uptake and completion, thus improving protection during high-risk periods, particularly for those who miss earlier opportunities to begin vaccination.

### 7.3. Opportunistic Vaccination Among Low-Adherence Populations

Immunogenicity and safety data clearly support the broader use of Encepur’s Express schedule beyond those needing immediate protection. As discussed, TBE vaccine uptake is low in many countries across Europe. Individuals with limited access to healthcare, irregular medical engagement, or general vaccine hesitancy may be less likely to initiate or comply with TBE vaccine schedules, despite TBE risk. Arguably, this group of potential vaccinees represents a key demographic for whom elongated dosing schedules may be a practical barrier to vaccination. In such cases, the availability of an accelerated schedule, such as Encepur Express, could be of value. When vaccination opportunities arise among individuals who fall into this category (e.g., during occupational health visits, school campaigns, or point-of-care consultations), the ability to initiate and complete the full primary series within a short window may improve adherence. Indeed, studies in different disease settings have reported that poor vaccine compliance is often linked to the perceived inconvenience of complex and elongated vaccine schedules and the tendency for people to forget obligations associated with effort [2,31]. As such, HCPs and relevant advisory bodies may consider the Express schedule as a tool for reducing the likelihood of drop-offs between doses, thereby increasing the number of people completing the vaccination series, thus improving TBE prevention.

## 8. Discussion

As the endemicity of TBEV is increasingly recognised across Eurasia, vaccination remains the most effective means of prevention. For either of the available European TBE vaccines, Encepur or FSME-Immun, receipt of two doses elicits strong immune responses and is associated with meaningful short-term protection. However, completion of the three-dose primary immunisation schedule is crucial for establishing durable protection against TBE, which can be sustained for periods of at least 10 years [22,37]. Despite this, both vaccine coverage and series completion remain insufficient across Europe [27,29,30,31,32]. The implications of this are incomplete and/or waning protection among individuals and insufficient immunity at the population level. As such, improving vaccine coverage and ensuring completion of the full primary immunisation series are essential for TBE prevention.

Encepur’s unique Express schedule, whereby all three priming doses are administered within just 21 days, offers a potential solution. As discussed throughout, the safety profile associated with the Express regimen is as expected for Encepur, with no additional safety concerns related to the expedited schedule or its subsequent booster regimen [37,42]. Furthermore, current data in both adults and children demonstrate that the immunogenicity of Encepur’s Express schedule is comparable to that of the Rapid or Conventional schedules for either vaccine [16,17,40,41]. An additional advantage of the Express schedule is that maximum seroconversion is achieved just six to seven weeks after initiating vaccination, as opposed to 5–12 months later with other dosing schedules [16,17]. Furthermore, as antibody responses are still detected up to and beyond Day 300 following primary vaccination [16,17,40,41], and can be maintained for up to 15 years with a first booster, protection appears to be durable [53].

In summary, while both Encepur and FSME-Immun have previously demonstrated comparable immunogenicity and safety when administered according to their approved schedules, Encepur’s Express schedule may be particularly useful for individuals who need rapid protection, those initiating vaccination late in the season, or groups at risk of incomplete series completion. Thus, the choice of vaccine and schedule should be guided by availability, cost, and local recommendations, as well as individual patient circumstances. Where appropriate, wider application of the Express schedule could help support timely completion of the primary series and ensure protection before peak TBE risk periods.

## Figures and Tables

**Table 2:** Current TBE vaccine booster schedules.

	Encepur		FSME-Immun	
Primary Dosing Schedule	First Booster	Subsequent Boosters	First Booster	Subsequent Boosters
Conventional	3 years after last primary dose	Every 3–10 years ^a^	3 years after last primary dose	Every 3–5 years ^b^
Rapid ^c^	3 years after last primary dose	Every 3–10 years ^a^	3 years after last primary dose	Every 3–5 years ^b^
Express ^d^	12–18 months after last primary dose	Every 3–10 years ^a^	-	-

^a^ Every 5 years in <12-year-olds, every 5–10 years in those aged 12–49 years old, and every 3 years in >49-year-olds; ^b^ Every 5 years in those aged 16–60 years old, and every 3 years in those >60 years old; ^c^ Formerly known as “Accelerated Conventional” for Encepur; ^d^ Formerly known as “Rapid” for Encepur.

## Data Availability

Not applicable.

## Abbreviations

ELISA	enzyme-linked immunosorbent assay
GMT	geometric mean titre
HCP	healthcare professional
NT	neutralizing antibody titres
TBE	tick-borne encephalitis
TBEV	tick-borne encephalitis virus
VE	vaccine effectiveness
